# Protocol for a prospective mixed-methods longitudinal study to evaluate the dynamics of contraceptive use, discontinuation, and switching in Kenya

**DOI:** 10.1186/s12978-019-0797-3

**Published:** 2019-09-05

**Authors:** Susan Ontiri, Lilian Mutea, Maxwell Muganda, Peter Mutanda, Carolyne Ajema, Stephen Okoth, Solomon Orero, Ruth Odhiambo, Regien Biesma, Jelle Stekelenburg, Mark Kabue

**Affiliations:** 1 Jhpiego Corporation, An affiliate of Johns Hopkins University, Nairobi, Kenya; 2 USAID Kenya and East Africa, Nairobi, Kenya; 3 Department of Health Sciences, Global Health, University Medical Centre Groningen/University of Groningen, Groningen, The Netherlands; 4 Department of Obstetrics and Gynecology, Leeuwarden Medical Centre, Leeuwarden, The Netherlands; 5 Jhpiego Corporation, An affiliate of Johns Hopkins University, Baltimore, MD USA

**Keywords:** Contraception, Discontinuation, Switching, Interventions, Side effects, Counseling, Quality of care, Study protocol

## Abstract

**Background:**

More women are accessing modern contraceptive use in Kenya, however, contraceptive discontinuation has stagnated over the decades. Any further increase in contraceptive use will most likely be from past users, hence understanding the dynamics of discontinuation while addressing quality of family planning services offered at health facilities and communities is critical for increasing the contraceptive prevalence rate and reducing the unmet need of family planning. The paper presents a study protocol that intends to evaluate the dynamics of contraceptive use, discontinuation, and switching among women of reproductive age initiating use of a contraceptive method.

**Methods:**

This longitudinal mixed-methods study is being conducted in Migori and Kitui counties, Kenya. A formative assessment using Interviews with adolescents, older women, heterosexual couples, health care workers, and community health volunteers explored barriers to contraceptive continuation and perspectives on discontinuation utilizing a qualitative cross sectional study design. Following the formative assessment, a client-centered intervention focusing on improving quality of family planning services, including counseling, will be implemented in 10 health facilities. A 24-month prospective cohort study among women of reproductive age initiating contraception with follow-up at 3, 6, 12, and 24 months will then be undertaken to assess the discontinuation rates, examine the dynamics of contraceptive use, discontinuation and switching, and further explore barriers and enablers for contraceptive continuation and switching among the study population.

**Discussion:**

In sub-Saharan Africa, contraceptive discontinuation studies have mainly been based on survey data that is collected retrospectively. By implementing a longitudinal mixed-methods study, we gain deeper insights into the contraceptive dynamics influencing the decision to continue, discontinue, and even switch following implementation of a client-centered intervention that enhances quality of care. Additionally, the study will shed more light on the profile of women discontinuing contractive use and further explore individual and couple-level dynamics influencing decision-making on continuation and discontinuation. The findings of this study will provide information that can be used to develop and implement human-centered interventions that focus on improving quality of family planning services and consequently improved continuation rates and overall satisfaction with method.

**Trial registration:**

The study is registered with the Clinical Trials Registry, NCT03973593.

## Plain English summary

Effective use of family planning can reduce maternal and child mortality. Globally, emphasis has been placed on increasing the pool of contraceptive users with no corresponding effort in ensuring that current users are continue to be satisfied with their methods and in improving the quality of family planning services, which would reduce discontinuation while users are still in need of contraception. Contraceptive discontinuation while still in need increases a woman’s risk of unintended pregnancy and is an important public health concern. This paper presents a protocol that aims to understands the dynamics of contraceptive use, discontinuation, and method switching. We will implement a family planning intervention that focuses on improving the quality of family planning services, with an emphasis on effective counseling in health facilities and communities. In the formative stage of the study, we will carry out qualitative interviews with women and couples reporting discontinuation while still in need and with health care workers and community health volunteers providing family planning services to understand their perspective and experience with contraceptive discontinuation and method switching. The study will also recruit 1016 women of reproductive age initiating a contraceptive method in 10 health facilities and enroll them in a 24-month study period with follow-up at 3, 6, 12, and 24 months. Upon completion, we will provide information to better understand factors and reasons associated with discontinuation and strategies for implementing contextualized client-centered interventions that focuses on improving satisfaction with contraceptive methods resulting to a reduction in the discontinuation rates.

## Background

Contraception use is a key, cost-effective public health interventions with the ability to reduce maternal mortality by 30% and child mortality by 10%, mainly by reducing the number of unplanned pregnancies, hence, the importance of fulfilling the unmet needs for family planning [1]. Global emphasis has been placed on improving contraception uptake with the FP2020 target of having 120 million additional women and girls in 69 of the world’s poorest countries by the year 2020, which was inspired by the 2012 London summit on family planning [2]. The focus on additional users underscores the need for family planning programs to ensure that clients have access to contraceptive methods and services so they can remain users for as long as they want to delay or avoid pregnancy [3].

Despite the significant benefits that contraception confers, not all women use methods consistently when still in need. Contraceptive discontinuation entails abandonment of a family planning method while at risk of an unintended pregnancy, and is a serious public health concern since it is associated with negative reproductive health outcomes. In an analysis of 38 Demographic and Health Surveys (DHS) from 34 countries globally, one out three women reporting unintended pregnancies were past contraceptive users who had discontinued use of contraception [4]. Contraceptive discontinuation occurs for various reasons; the majority of women who discontinue while in need of contraception have cited non-method and method-related concerns, including failure and side effects, as the primary reasons for discontinuation [5]. Health system factors, including poor service quality, lack of adequate method mix and choice, commodity stock-outs, and weak referral system, have also been cited as drivers for discontinuation [4].

Globally, 44% of all pregnancies are unintended with approximately half of these ending up in abortion [6]. Previous studies have found contraceptive abandonment contributed substantially to the continued high total fertility rate, unwanted pregnancies, and induced abortions [7]. Most family planning programs have been designed to increase the number of users. Few programs assess the level of users’ satisfaction with contraceptives in general and with the current method in particular because these women have already overcome the initial well-known barriers of family planning use—distance, cost, and fear of side effects [8].

Kenya’s modern contraceptive use among married women is 53%, with an unmet need of family planning of 18% and a total fertility rate of 3.9, as per the last DHS of 2014 [9]. The more recent PMA 2020 round 7 data from 2018 indicates a further increase in the modern contraceptive prevalence rate (mCPR) to 60.7% and a reduction in unmet need to 13.8% [10]. Despite the improvement in contraceptive use, discontinuation rates have remained higher over the last three decades with one out three women discontinuing use by 12 months [9]. Contraceptive continuation rates has been suggested as a useful summary measure of the overall effectiveness of program services in enabling clients to sustain contraceptive use even though they may switch from one method to another [11, 12]. The PMA 2020 round of surveys have shown that there is still a huge gap in provision of quality of family planning services at the health facility and community levels, with only 67% of the women informed of other methods, 59% counseled on side effects, and 54% told what to do when they experience the side effects, thus presenting missed opportunities for provision of quality family planning services that might promote continuation or method switching [10].

In Kenya, as in other lower-middle income countries, the national health management information system does not monitor contraceptive continuation at the facility level, as the data capture mechanism is not longitudinal. More so, within the health system, only users of reversible methods, including implants and IUDs, go back to a facility, since they require a clinical intervention during removal of these methods. Users of short-term methods, like condoms, pills, and injectables, simply fail to turn up for their next appointment for resupply. Therefore, tracking contraceptive discontinuation and method switching is limited to use of survey data such as a DHS that collects calendar data on women’s history of contraceptive use.

There is overwhelming evidence regarding benefits of contraceptive use, including strategies that programs can undertake to improve uptake. However, there is limited knowledge of the determinants of discontinuation and switching of contraceptive methods, a gap that the World Health Organization has identified, hence listing contraceptive discontinuation and method switch as one of the global family planning research priorities [13]. To address this among other health outcomes, the United States Agency for International Development (USAID) Kenya and East Africa funded a 5-year reproductive maternal, newborn, child, and adolescent health; nutrition; and water, sanitation, and hygiene project, named Afya Halisi*,* that is being implemented in four counties in Kenya. This longitudinal study will be anchored within the Afya Halisi project, which seeks to scale up high-impact interventions and practices to improve the quality of family planning services.

### Overview of the family planning quality improvement project

The family planning quality improvement component of the wider maternal, newborn, child, and adolescent health project is built on the underlying principle of provision of respectful health care for all clients. The focus of the family planning component of this health system strengthening project is to increase access and improve the quality of family planning services offered at facilities and to strengthen the community health service delivery that will ensure meaningful male involvement in family planning. This includes providing support to the health facilities to address the six fundamental elements of quality of family planning services: choice of contraceptive methods, information given to clients, technical competence, interpersonal relations, follow-up/continuity mechanisms, and appropriate constellation of services as articulated by Bruce’s quality framework [14].

The various aspects of the intervention are as follows:

#### Equipment and supply chain

- Availability of a consistent supply of family planning commodities and essential equipment and supplies at health facilities to enable individuals and couples to have an adequate supply of their preferred method.

#### Capacity building

- Capacity building of health care workers on the provision of family planning through mentorship and coaching to augment the knowledge and skills they have gained from previous class room trainings. This is in line with study findings that show mentorship and coaching are effective strategies for reinforcing learning processes, improving provider and manager motivation, and improving clinical performance [15, 16]. Provision of training models to project supported facilities will allow health care workers to sharpen their skills even in the absence of clients.
- Strengthening the quality of contraceptive counseling offered by health care workers by adapting Kenya’s balanced counseling strategy by co-creating key messages on contraceptive continuation and switching with providers, community health volunteers, and women. Health care workers are coached on how to offer client-centered individualized and partner counseling within the parameters of respectful care. This is a follow-up of the counseling and referral done by community health volunteers. Emphasis is placed on strengthening contraceptive counseling, especially on side effects and what women and couples should do when they experience side effects.
- Capacity building of community health volunteers in developing family planning male champions in the communities. Small group dialogue meetings with men are held to gain insights into their definition and interpretation of male involvement in family planning programs. The forums are used to dispel myths and misconceptions on family planning among men and encourage spousal support in decision-making.

#### Service delivery channels

- In Kenya, most facilities provide family planning methods during morning hours from Monday to Fridays. To encourage adolescents and youths to access contraceptive services, the project will strengthen youth-friendly services in the facilities by conducting whole site orientation for all staff working in the facilities and by extending service provision to weekends.
- Scale up the implementation of community-based distribution of contraceptives, including pills, condoms, and injectables, that are allowed within Kenya’s task-sharing guideline.

#### Community level

- Strengthen community-facility linkage through the use of community health volunteers who track women and check on their progress routinely, referring them back to health care workers for additional support when and if needed. The project will institute a tracking mechanism that will enable providers to keep track of women on short-term methods to ensure that prompt follow-up is done for their resupply, within the grace period, to prevent unintended pregnancies.
- Capacity building of community health volunteers linked to the study facilities to enhance their knowledge on family planning, including counseling. The current training on community-level family planning services focuses on general information about each contraceptive method but lacks adequate content on counseling for family planning. The community health volunteers’ training package is enhanced with additional content on contraceptive methods that will equip them with the necessary skills on follow-up, counseling for side effects, and referrals where necessary.

## Study aim

In this paper, we describe a protocol for an evaluation of contraceptive continuation and method satisfaction among women and couples following interventions that are geared toward reducing discontinuation by improving the quality of family planning services offered at both health facilities and community levels. This prospective study is expected to further increase the understanding of the dynamics of decision-making among individuals and couples that influence either contraceptive continuation or discontinuation. Understanding the factors that affect the discontinuation of contraceptive use is crucial to ensuring that women and couples can attain their long-term fertility desires and have desirable reproductive health outcomes, including reducing maternal and child mortalities.

The study objectives are:

1. To explore reasons and factors leading to contraceptive discontinuation
2. To determine the contraceptive discontinuation rates at 3, 6, 12, and 24 months among women of reproductive age following a health system strengthening project that is focusing on improving quality of family planning services
3. To investigate predictors of contraceptive discontinuation and switching
4. To understand barriers and enablers for contraceptive continuation and switching

## Methods

### Study setting

The study will be implemented in 10 sites, both public and private health facilities in Migori and Kitui counties in Kenya. Migori is in southwestern Kenya, 368 km from Nairobi, Kenya’s capital, and borders Tanzania to the west. Kitui is 170 km south-east of Nairobi. Kenya’s population is estimated to be 51 million people, Migori and Kitui counties have a population of 1.1 million each. The total fertility rate for Migori and Kitui counties is 5.3 and 3.9 respectively [9]. As per the last Kenya DHS of 2014, the mCPR for Kitui County was 55%, two percentage points higher than the national average; the recent PMA 2020 data of 2018 indicated an improvement to 63% [9, 10]. Migori’s 2014 mCPR was 44%, nine percentage points lower than the national average [9]. The two counties have a diverse method mix; Migori’s mCPR is mostly driven by long-acting reversible contraceptives, at 72%. In Kitui, short-term methods are more popular, at 64%. While there is no ideal method mix, the study will explore the dynamics and drivers of contraceptive use in the two counties, which have a varied method mix, to assess how they influence discontinuation or continuation.

### Study design

This single group longitudinal study is being implemented in two phases employing multiple designs to respond to the objectives.

#### Phase 1: formative assessment

Information on objective 1 of the study was gathered through a formative assessment that utilized a qualitative study design. The aim of the formative assessment is to explore the reasons for discontinuation, including barriers to contraceptive continuation and switching. Qualitative interviews were done with both men and women reporting contraceptive discontinuation with unmet need of family planning. The interviews focused on understanding the reasons for discontinuation and identifying strategies that can promote contraceptive method satisfaction and continuation rates. Interviews with health care workers and community health volunteers were conducted to gain further insights into contraceptive discontinuation and barriers to provision of quality family planning services. The information gathered will be used to further refine the family planning quality improvement project by using human-centered design to develop context specific strategies that are client centered. The focus will be on implementing a client-centered counseling approach delivered by both health care workers and community health volunteers to improve the quality of the family planning services and promote contraceptive continuation among women and couples.

##### Sample size and sampling strategy

Purposive sampling was used to select participants for the qualitative study from the catchment population of the 10 study facilities. The qualitative study included six key informant interviews with reproductive health and community strategy coordinators, 10 in-depth interviews with adolescents, 10 in-depth interviews with married, heterosexual couples, and 16 focus group discussions with adolescent mothers, women aged 20–49 years, health care workers, and community health volunteers. An estimated 196 participants participated in qualitative data collection in total. Eight to 10 participants were invited to participate in each focus group discussion; key informant interviews and in-depth interviews were one-on-one.

##### Data analysis

Data from focus group discussions, key informant interviews, and in-depth interviews will be translated and transcribed in English then entered into qualitative data software (Atlas ti.8). Independent coding will be conducted by different analysts and subsequent comparison between the developed coding matrices will be used for developing a reliability factor for the analysis. A coding frame will then be developed using a grounded theoretical approach. The trends from the emerging themes will be iteratively developed by repeatedly analyzing data collected from participant categories. Verbatim text generated alongside the code matrix will be used to support the emerging thematic framework. A theoretical framework for the underlying reasons for contraceptive discontinuation and strategies for promoting continuation and improved quality of family planning services among various population segments will then be developed.

#### Phase 2: evaluation of the prospective cohort

A prospective cohort design will be utilized in this phase among women initiating family planning services in 10 study sites in Kenya.

##### Study population and eligibility criteria

Women of reproductive age, 15 to 49 years initiating family planning services in 10 health facilities in Kitui and Migori counties are eligible to participate. All enrolled individuals will be invited for follow-up interviews at 3, 6, 12, and 24 months. The following will be excluded from participation in this study: women who take up condoms, emergency contraception, or voluntary surgical contraception; are not residents of the selected study region; women do not have access to a phone they can control for ease of follow-up and are not willing to be followed up for a period of 24 months with four contacts; and women who are planning to get pregnant within the study duration.

##### Participant recruitment

Trained health care providers who offer services at family planning clinic will identify clients who are willing to participate in the 24-month prospective cohort evaluation. Participants will be recruited after receiving a family planning service either at the family planning, maternity, or HIV clinic. The health care worker will inform them of the ongoing study on dynamics of contraceptive use. The provider will then link women who are interested in hearing more about the study with the research team, who will be stationed at the facility. The research assistants will explain the details of the cohort study, guided by a brief recruitment script to confirm eligibility of the study, and if interested, obtain their permission for additional screening and consenting. During the additional screening and consenting process, potential participants will receive additional information about the follow-up phone interviews at 3, 6, 12, and 24 months after baseline and inquire if they are still interested in participating in the study. The research assistant will document the phone number for a follow-up phone interview; this is a part of the consent process. Written consent will be obtained and those who are illiterate will make a thumb print impression on the space designated for the signature on the consent form. Recruitment is expected to take approximately 3 to 4 months.

##### Participant follow-up

**Fig. 1:**
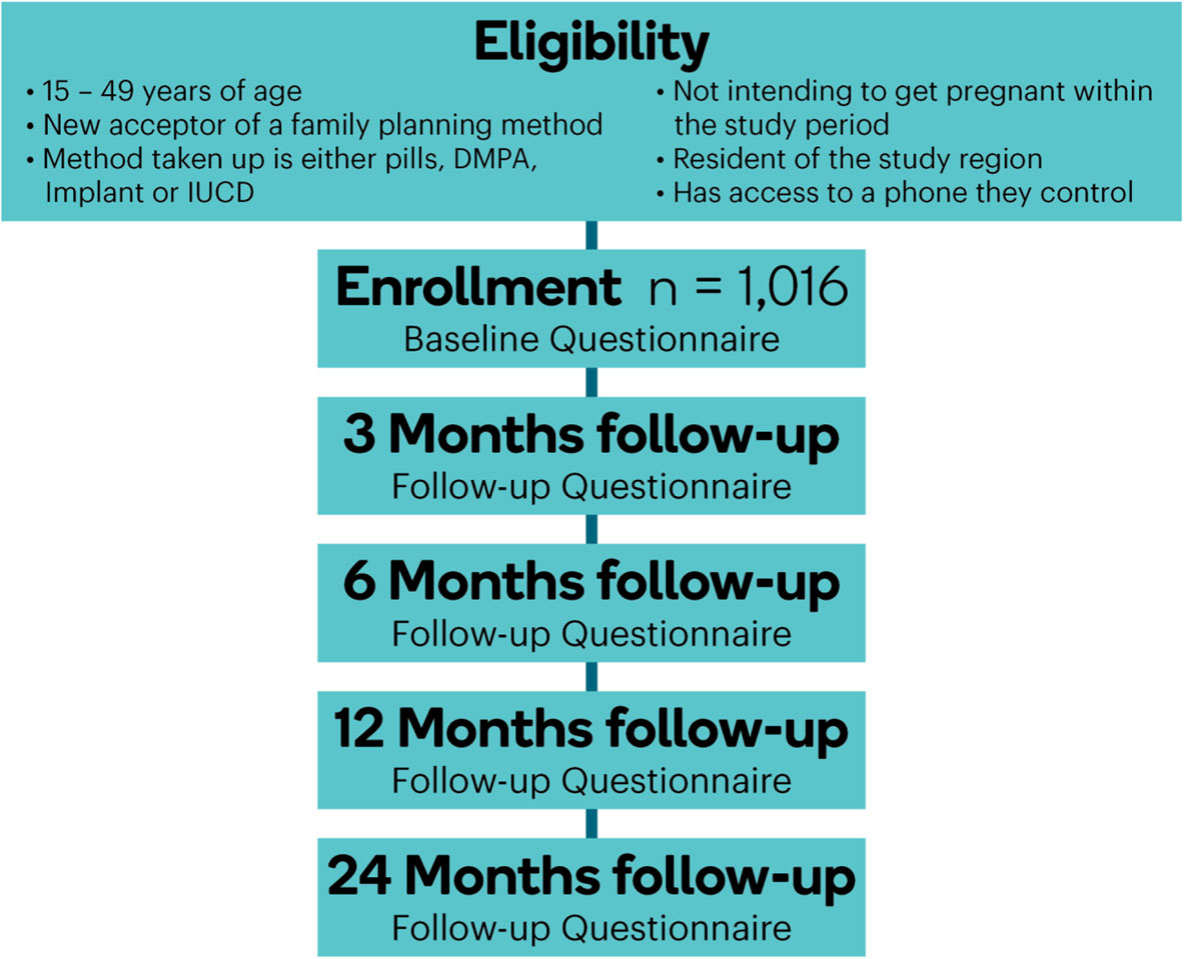
Flow chart of the prospective cohort study

Participants will be enrolled for 24 months. Data on current method use, side effects and complications, fertility intentions, satisfaction, motivation, and decision-making, including male partner involvement on contraceptive use, will be collected at five different points in time to assess the dynamics of contraceptive use, either continuation, discontinuation, or switching. Data collection will occur the following time points: at baseline and at 3, 6, 12, and 24 months (Fig. 1). At baseline, individuals who are eligible to participate and have consented to be part of the study, will complete an online questionnaire, which will be more extensive (the questionnaire is described in more detail below), prior to leaving the health facility. Participants will provide their phone contact information to enable the research team to get in touch in the subsequent follow-up period for brief interview sessions. During each follow-up interview, consent will be sought to ensure the participants are still interested in participating in the study. During each round of follow-up interviews, three attempts will be made to contact participants before a determination of loss to follow-up is made. The second and third round of interviews will take place at 3 and 6 months after baseline, because women who choose some short-term methods will require a 3-month cycle for resupply. The follow-up at 12 months will be used to determine the contraceptive continuation rate, which is the primary outcome for this study. The 12 and 24 months’ period allows comparisons with other surveys, including the DHS, that have used the same time reference to report discontinuation.

##### Questionnaire

The baseline questionnaire has five sections: the first section covers socio-demographic characteristics, section two is on pregnancy and fertility preferences, section three is on contraception services received, section four is on the perception of the quality of care received at the health facility, and the section five is on the decision-making environment. From the pilot study conducted, we anticipate initial data collection to take 20–25 min. The follow-up questionnaires are shorter with only two sections; the first section is on contraceptive history. Participants will be asked whether they are still using their baseline contraception method, any side effects and or complications experienced, and the level of satisfaction with the method. The second section will be for clients reporting discontinuation or contraceptive switching, both closed- and open-ended questions on the timing and reasons for discontinuation or switching, including fertility intention, will be asked.

##### Sample size

This phase is powered to detect a 10 percentage point difference in the primary outcome of interest— reductions in contraceptive discontinuation rates at 12 months, between baseline and endline levels with a 95% confidence interval and a power of 80%. For the purpose of the sample size calculation, Kenya DHS data was used for the current contraceptive discontinuation rate of 31% [9]. Further, the sample size is adjusted to account for a 30% attrition rate due to potential dropouts and loss of clients during follow-up through the four waves of data collection up to 24 months and a design of effect of 1.5 due to cluster sampling. This results in a total sample size of 508, which is doubled for the overall study (for the two counties), leading to 1016 women. Proportionate to size is used to allocate the sample size for each of the 10 facilities based on the current family planning caseload. Additionally, the current method mix for the two counties further determines the sample for each modern contraceptive method.

##### Data quality assurance mechanisms

At enrolment, quantitative data will be collected by 10 research assistants with a minimum of a diploma degree. The research assistants undergo a 5-day training on data collection, which will focus on basic research ethics and study procedures.

##### Outcomes

The primary outcome of this study is the contraceptive discontinuation rate among the family planning users at 12 months. Participants will be considered to have discontinued if they report not using their baseline method during the follow-up interviews. Participants who discontinue use of the method used at the time on enrollment and initiate use of different method within one month will be considered to have switched methods. Other secondary outcome measures include discontinuation at 3, 6, and 24 months; the method information index [11] will be used as a proxy for quality of family planning counseling.

##### Data analysis

Quantitative data will be collected on RedCap [17] and cleaned before analysis to ensure completeness. Descriptive analysis of quantitative variables will be done using measures of central tendency (mean, median) and measures of dispersion (range, standard deviation), as appropriate. Descriptive statistics will be used to assess contraceptive discontinuation, including switching. Pearson’s χ2 or t test, where appropriate, will be used for univariate analyses to compare characteristics of women who continued and discontinued their initial contraceptive method. Multivariate analysis will be implemented for variables that will be significantly associated with contraceptive continuation at bivariate analysis. The time to event for this survival analysis will be calculate from method initiation to the time point when the participant reports discontinuation with her baseline method. Censoring will be done for clients lost to follow-up and those reporting discontinuation of their baseline method. Contraceptive discontinuation rates will be calculated using the Kaplan Meier survival function. Cox proportional hazard regression model will be used to estimate hazard ratio for risk of contraceptive discontinuation for independent predictors of discontinuation. The level of significance will be set at a *p*-value < 0.05. For qualitative data, the approach for data analysis will be similar to what has been presented above under the formative assessment.

##### Dissemination of study findings

The study team plans to disseminate findings among national and sub-national stakeholders through in-country dissemination events to demonstrate how, with a focus on quality in family planning, the level of satisfaction and discontinuation rate have changed. The results will be published in peer-reviewed journals and presented at both national and international conferences. Briefs indicating policy and programmatic implications will be developed and shared with the government and other stakeholders. The study will contribute to the body of knowledge that will inform decision-makers locally and globally on the determinants of contraceptive discontinuation and couple-level decision-making on the same.

### Ethical considerations

Written informed consent will be obtained for all study participants. All married adolescents and those who have previously given birth will be considered emancipated/mature minors and eligible to give consent themselves. All respondents will be informed about the objectives, procedures, benefits, and risks of the study through the process of obtaining informed consent. There is some minimal risk associated with participating in the prospective cohort study especially among women taking a family planning method without the knowledge of their partners, hence, follow-up interviews might expose them. Information regarding the follow-up interview will be communicated to the participants at the time of consenting during enrolment. Participants will be asked whether they will have any concerns about their privacy regarding the follow-up interviews. During the follow-up interviews, the research assistants will establish whether the participants are in a safe environment where they can talk freely. The English questionnaire will be translated into and back-translated from Dholuo for Migori, Kamba for Kitui, and Swahili for both counties. All research assistants engaged in this study will be recruited based on prior experience in administering questionnaires. They will be trained on study design and procedures and ethical consideration, including the need for confidentiality, anonymity, consent, as well as their role as data collectors and how to complete the data collection tool. Field testing of the tool among a similar group of study participants will be undertaken prior to the study to identify potential issues that needs modification. Monitoring visits will be made by the principal investigator and co-investigators to ensure fidelity to the study protocol.

### Study status

Data collection for the formative assessment was done in May 2019 and the analysis of the results is ongoing. Refining the quality of family planning interventions using a human-centered design methodology will be done after July 2019. Enrollment of prospective cohort participants and collection of baseline data is scheduled to begin in October 2019.

## Discussion

The current evidence suggests that discontinuation can be reduced by strengthening quality of services and meeting women’s reproductive health needs [18, 19]. By implementing a project that aims on improving service delivery, some of the health system barriers that lead to discontinuation might be removed. For a country like Kenya that has seen a tremendous growth in contraceptive use and its current position in the family planning S curve [20] of high prevalence and latent growth, further growth in the mCPR modern contraceptive prevalence rate and a corresponding reduction in unmet need of family planning will likely stem from past users who have discontinued while in need of contraception.

Most studies in contraceptive discontinuation have relied on survey data based on DHS calendar information that is collected retrospectively hence prone to recall bias. By implementing a prospective cohort study, we gain deeper insights on discontinuation and method switching. Additionally, the study will shed more light on the profile of women discontinuing and further explore individual and couple-level dynamics influencing continuation and discontinuation. The information to be collected has the potential to provide empirical evidence for strategies to promote interventions that focus on improving quality of family planning and consequently improved continuation rates and satisfaction with method.

## Data Availability

Not applicable

## Abbreviations

DHS: Demographic and Health Survey
mCPR: modern contraceptive prevalence rate
USAID: United States Agency for International Development
